# The Influence of Dietary and Physical Exercise Habits on Melanoma Risk: A Case–Control Study

**DOI:** 10.3390/nu18121919

**Published:** 2026-06-12

**Authors:** Francesca Crespí-Payeras, Rosa Moll-Amengual, Neus Calbet-Llopart, Judit Mateu, Míriam Potrony, Cristina Carrera, Pablo Iglesias, Gemma Tell-Martí, Teresa Torres Moral, Susana Puig

**Affiliations:** 1 Melanoma Unit, Universitat de Barcelona (UB), 08007 Barcelona, Spain; fjcrespi@recerca.clinic.cat (F.C.-P.); potrony@clinic.cat (M.P.); ccarrera@clinic.cat (C.C.);; 2 Centro de Investigación en Red de Enfermedades Raras (CIBER-ER), Instituto de Salud Carlos III, 08036 Barcelona, Spain; 3 Fundació de Recerca Clínic Barcelona-Institut d’Investigacions Biomèdiques August Pi i Sunyer (IDIBAPS), 08036 Barcelona, Spain; mateu@recerca.clinic.cat (J.M.); piglesia@recerca.clinic.cat (P.I.); tetorres@recerca.clinic.cat (T.T.M.); 4 Melanoma Unit, Dermatology Department, Hospital Clinic de Barcelona, 170 Villarroel, 08036 Barcelona, Spain; 5 Biochemistry and Molecular Genetics Department, Hospital Clinic de Barcelona, 170 Villarroel, 08036 Barcelona, Spain; 6 Pharmacovigilance Executive Committee, Hospital Clinic de Barcelona, 170 Villarroel, 08036 Barcelona, Spain

**Keywords:** melanoma, body mass index, dietary habits, physical exercise

## Abstract

Background/Objectives: Obesity, food and nutrient intake, and physical activity (PA) have been linked to the occurrence of various types of cancer. However, evidence regarding their relationship with melanoma is limited. We aimed to assess whether body mass index (BMI), diet quality, food cooking methods, and PA influence the risk of developing melanoma. Methods: This case–control study compared the demographic characteristics, dietary habits, and PA of 130 melanoma patients from the Hospital Clínic de Barcelona with 166 control subjects of similar age and sex distribution. Data was collected by means of a questionnaire, administered between January 2016 and February 2020. The association between these factors and melanoma was assessed using odds ratios for binary variables with 95% confidence intervals. Results: BMI was not found to be associated with the diagnosis of melanoma. However, restricting foods and limiting sugary products did show a correlation with lower melanoma risk, while dairy product restriction was associated with an increased risk. Consumption of processed meats and unhealthy cooking methods were also associated with an increased risk of melanoma development. Lastly, an inverse association between PA practice and frequency and melanoma risk was observed in women, while vigorous-intensity PA showed an inverse association regardless of sex. Conclusions: This study identifies specific dietary patterns and PA behaviors that may play a role in melanoma risk, highlighting the potential for personalized lifestyle-based prevention strategies.

## 1. Introduction

Melanoma is a multifactorial disease influenced by the interplay of genetic, phenotypic, and environmental factors, with ultraviolet radiation (UVR) exposure recognized as the primary environmental risk. In recent years, growing attention has been directed toward modifiable lifestyle factors, particularly diet, physical activity (PA), and body composition, as potential contributors to melanoma susceptibility and progression [1]. According to the American Institute for Cancer Research (AICR) and the World Cancer Research Fund (WCRF), up to 40% of all cancers could be prevented through healthier dietary habits, regular PA, and weight control [2,3]. Indeed, epidemiological data indicate that a poor-quality diet [4], physical inactivity, and being overweight or obese not only promote cardiovascular diseases but have also been identified as significant risk factors in a range of malignancies [2,5].

Although the role of nutrition and PA in other skin pathologies and in cancer prevention is well-established [6,7,8], evidence specific to melanoma remains limited and, not infrequently, contradictory. Recent research has identified several foods and nutrients with potentially protective effects in the context of melanoma [9]. A recent review by Dong et al. showed that the intake of foods rich in vitamins A, C, and E, carotenoids, and coffee may provide a protective effect due to their antioxidant properties which combat free radicals produced by UVR [10]. Moreover, consumption of a fiber-rich diet may have a protective effect against melanoma as well as on immunotherapy responses [10,11]. These claims, though, remain controversial as the effects and biological mechanisms of most nutrients on melanoma risk are not fully understood [1,10]. Conversely, alcohol and products rich in sugars and carbohydrates (high glycemic load) have been associated with an increased risk [12,13], along with high energy intake and saturated fat consumption [14]. Overall, the WCRF and the AICR recommend a diet rich in vegetables and whole grains, but low in red meat, processed foods, and refined carbohydrates [3].

While PA has been consistently associated with a reduced risk of several cancers and even a reduction in mortality rates [5], it has been suggested that PA may increase the risk of melanoma, likely related to greater sun exposure during outdoor activities. However, recent evidence points to the beneficial role of exercise in both melanoma prevention and progression [15], not only by modulating systemic inflammation and immune function, but also by influencing the tumor microenvironment and response to therapy [16,17].

Together, this data highlights the need for further research to define the influence of diet, body weight, and PA on melanoma development, with the goal of determining new prevention strategies for this disease.

Although obesity has been implicated in the development and progression of several malignancies, its role in melanoma is unclear: the consumption of specific foods could either increase the risk or have a protective effect. A number of epidemiological studies have associated regular PA with a lower risk of cancer development, but a causative relationship between PA and melanoma has not been established. By improving overall health, PA could well have a protective effect against the development of melanoma.

In this context, the study aimed to evaluate how body composition, dietary patterns, cooking practices, and PA impact on melanoma risk, so as to identify lifestyle factors that may contribute to prevention. Our findings highlight both several protective and harmful behaviors, underscoring the relevance of personalized, evidence-based strategies for melanoma prevention.

## 2. Materials and Methods

### 2.1. Study and Questionnaire Design

We conducted a case–control study involving 130 melanoma patients from the Melanoma Unit of the Hospital Clínic de Barcelona (HCB) and 166 healthy individuals, representative of the same Spanish population.

The data was obtained using a questionnaire on various aspects of diet and PA (Supplementary Material S1), adapted from the PREDIMED study [18], although the questionnaire was not formally validated. Melanoma patients completed the questionnaire at melanoma diagnosis, between January 2016 and February 2020, and the control cohort was obtained by distributing a digital format questionnaire (Google Forms) randomly among the general population during February 2020.

The questionnaire included the following sections: epidemiological characteristics (age, sex, weight, and height); omission of meals and food restrictions; weekly consumption of foods from a predetermined list; most common cooking methods for these foods; and the frequency of PA practice of different intensities during the previous week. The control version included a question regarding the existence of a personal history of melanoma so as to exclude such cases from the analysis.

### 2.2. Participant Selection

A total of 130 patients diagnosed with melanoma were prospectively included at HCB. They were all over 18 years of age and in different stages of the disease: 3.1% melanoma in situ, 15.4% stage I, 15.4% stage II, 43.9% stage III, 21.5% stage IV, and 0.8% with unmeasurable disease; 38% had active malignancy. All patients provided written informed consent.

**Figure 1:**
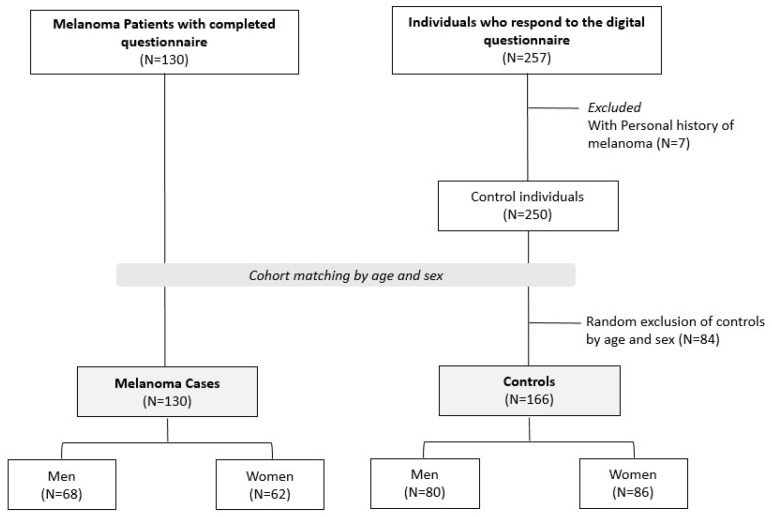
Participant selection flowchart.

The online questionnaire solicited 257 responses that were prospectively obtained from healthy individuals over 18 years of age. Seven individuals reporting a history of melanoma were excluded and, subsequently, eighty-four controls were removed to correct the age imbalance between groups, as the control group initially included many younger respondents. Ultimately, 166 healthy individuals were included in the control group. The flowchart of participant selection is shown in Figure 1.

### 2.3. Definition of Variables

**Figure 2:**
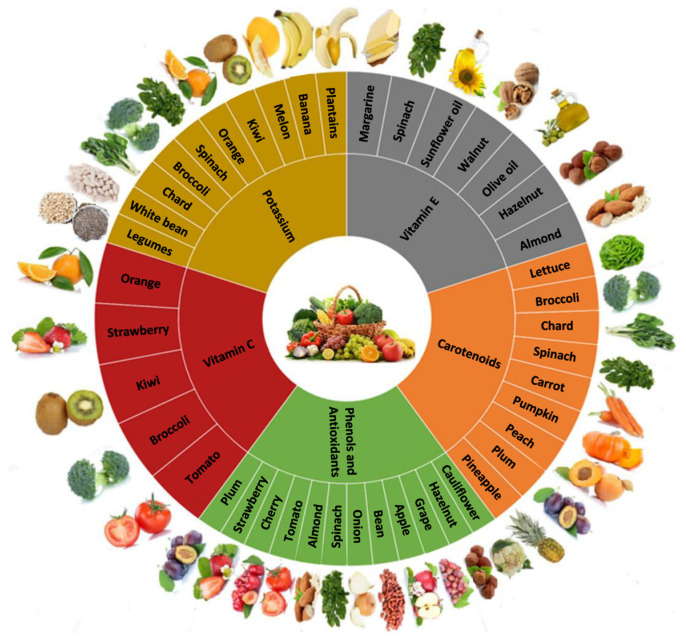
Classification of fruits and vegetables according to their predominant micronutrients.

The responses to the questionnaire were categorized into variables based on the existing literature. BMI (kg/m^2^) was categorized as “underweight” (<18.5), “normal weight” (18.5–24.9), “overweight” (25–29.9), and “obese” (>30). For the general aspects of diet, binary variables were created for “Omission of any meal” and “Restriction of certain foods”. Responses related to food restriction were further divided into five different binomial variables: “Dairy Products”, “Meat and/or Fish”, “Sugary Products” (pastries, pies, sugar, and sweets), “Cereals” (flours and gluten), and “Processed Products” (processed meats, fats, precooked and fast food). To quantify the consumption of specific foods three or more times per week, multiple binary variables were created, grouping various food items. Fruits and vegetables were categorized into five variables, according to their predominant micronutrients: “Vitamin C”, “Vitamin E”, “Carotenoids”, “Phenols and Antioxidants”, and “Potassium” [19] (Figure 2). Cereals were classified into “Fiber-rich Cereals” (>6 g/100 g) (e.g., whole-grain pasta, whole-grain bread, cereal bread, wheat flour, and cereals) and “Fiber-poor Cereals” (<6 g/100 g) (e.g., white rice, whole-grain rice, pasta, white bread, couscous, and semolina) [20], meat into “Red Meat” and “White Meat”, and fish into “White Fish” and “Blue Fish”. To analyze the most common cooking methods for each food, a dichotomous variable was created with the categories “Healthy” (raw, boiled, grilled, baked, and stewed) and “Unhealthy” (fried and breaded) [21].

Regarding PA, all data refers to the week prior to the completion of the questionnaire. Binary variables of “Physical Activity Practice” were described in general and according to two intensities: “Moderate intensity”, including, but not limited to, activities such as walking, dancing, and climbing stairs, and “Vigorous intensity” such as running, cycling, or swimming.

“General Physical Activity” was defined as any level of PA, regardless of intensity. To assess weekly activity patterns, we created variables for “Frequency of Physical Activity”, which were categorized into three groups based on the number of days per week that participants reported exercising: “0–1 days”, “2–3 days”, and “>3 days”. These frequency categories were applied separately to general activity, moderate-intensity activity (defined as ≥60 min per session), and vigorous-intensity activity (defined as ≥30 min per session).

### 2.4. Statistical Analysis

Continuous variables were tested for normality using the Shapiro–Wilk test. Student’s *t*-test and the Mann–Whitney–Wilcoxon test were used to compare continuous variables with normal and non-normal distributions, respectively. Categorical variables were compared using Pearson’s chi-square test. Univariable logistic regression was applied to evaluate the association between each independent variable and melanoma risk, expressed as odds ratios (OR) and 95% confidence intervals (95% CI). The same analyses were performed by stratifying by sex. To control for multiple comparisons, *p*-values were adjusted using the Benjamini–Hochberg false discovery rate (FDR). An adjusted *p*-value ≤ 0.05 was considered statistically significant. Analyses were conducted using complete-case approach, including only participants with available data for each variable. All analyses were performed using IBM SPSS Statistics version 25 software (IBM Corp., Armonk, NY, USA) and R software (version 4.5.0, R Foundation for Statistical Computing, Vienna, Austria).

## 3. Results

### 3.1. Baseline Characteristics

A total of 130 patients with melanoma and 166 healthy controls, comparable in age and gender distribution, were included (Table 1). BMI was compared between the two groups, and no statistically significant differences were found either between the medians (*p* = 0.805) or in the distribution of the different BMI categories (*p* = 0.648).

### 3.2. Dietary Aspects

Dietary habits were compared based on questionnaire data (Table 2). Meal omission was more frequent in controls, although this difference did not reach statistical significance (*p* adj. = 0.091). When stratified by sex, this difference showed a tendency towards significance in men (*p* adj. = 0.075), but not in women (*p* adj. = 0.729). Controls also restricted foods more often than melanoma patients (*p* adj. < 0.001). Dairy restriction was associated with a higher risk of melanoma (*p* adj. = 0.013), while the restriction of sugary products was associated with a lower risk (*p* adj. < 0.001), with similar findings observed in both sexes. Although not statistically significant after correction (*p* adj. = 0.072), cereal restriction in women showed a tendency toward a higher melanoma risk.

We also analyzed whether the consumption of specific foods or micronutrients more than three days a week could be associated with the development of melanoma (Table 2). It was observed that high consumption of processed meats (*p* adj. < 0.001) was associated with melanoma risk, especially in women. No further associations were evident after multiple-testing correction, and sex-stratified analyses showed no significant differences.

### 3.3. Most Common Cooking Methods

Regarding the association between the most common cooking methods for a range of foods (Table 3), it was observed that, in general, melanoma patients were more likely to use unhealthy cooking methods more frequently than control individuals. The effect was statistically significant for eggs (*p* adj. = 0.006) and white fish (*p* adj. = 0.028), with a clearly higher effect in men. For blue fish, the association was also significant (*p* adj. = 0.006), with a stronger effect observed in women. No differences were detected in the cooking methods for meat and seafood between patients and controls.

### 3.4. Physical Activity Practices

We analyzed whether general PA practice and its intensity could lower the risk of developing melanoma (Table 4). Analysis of all individuals showed no association between overall PA practice and the incidence of melanoma. However, when stratified by sex, it was observed that PA practice was more frequent in women in the control group than in melanoma patients (*p* adj. = 0.029). Similarly, when considering the intensity of PA, statistically significant differences were found for moderate PA practice in the female population (*p* adj. = 0.029). Notably, vigorous PA practice was more frequent in controls than in melanoma patients, regardless of sex (*p* adj. < 0.001), suggesting an inverse association between PA practice and intensity and melanoma risk.

Finally, we assessed whether weekly frequency of PA practices impacts the risk of developing melanoma (Table 4). When stratified by sex, this difference was pronounced and reached statistical significance in women (*p* adj. = 0.045).

## 4. Discussion

This study explored the relationship between BMI, dietary patterns, cooking practices, and PA in relation to melanoma risk in a population-based sample. The findings suggest that certain lifestyle-related factors may play a role in modulating melanoma susceptibility, complementing existing research on the influence of modifiable behavioral determinants in melanoma epidemiology.

Obesity is generally recognized as a risk factor for cancer development. However, the evidence regarding its role in melanoma remains controversial [22]. Recent studies have described an “obesity paradox” in melanoma, particularly in the context of immunotherapy, where overweight patients may experience improved treatment responses [17,23]. However, in the context of primary prevention, excess adiposity may contribute to systemic inflammation and immune dysregulation, potentially influencing melanoma susceptibility [24]. A recent study examining the impact of obesity-related dietary patterns on cancer incidence reported a strong association between obesity and several cancer types, including malignant melanoma [25]. Conversely, a meta-analysis found only a weak association between increased BMI and melanoma risk, although significant heterogeneity was noted among the included studies [26]. Other studies have reported no association between increased BMI and melanoma risk [27], nor with melanoma incidence [28]. In our cohort, BMI was comparable between cases and controls, suggesting no clear association with melanoma risk. This may reflect the limitations of BMI as a measure of adiposity, as it does not distinguish between fat distribution or metabolic status. Although emerging evidence points to visceral fat and inflammation as potentially more relevant factors in melanoma susceptibility and response to immunotherapy [24,29,30], these variables were not directly assessed in our study. Therefore, our findings do not allow us to explore these mechanisms, and interpretations regarding adiposity-related pathways should be made with caution. Future studies using more precise body composition metrics may help clarify these associations. Beyond BMI, emerging evidence highlights the role of specific dietary patterns in cancer prevention and treatment. The Mediterranean diet (MD), intermittent fasting (IF), and calorie restriction (CR) have gained attention for their potential to modulate systemic inflammation, enhance immune function, and improve metabolic health. The MD, widely considered as one of the healthiest dietary patterns due to its richness in antioxidants and anti-inflammatory nutrients, has been associated with reduced cancer [31], including protective effects in melanoma [32,33]. Moreover, IF has been shown to promote the growth of beneficial gut microorganisms with anticancer properties [34]. Although recent studies have provided more evidence supporting IF and CR as cancer prevention strategies [22], only the MD is the dietary pattern currently recommended by the ACRI for cancer prevention [34].

In our cohort, food restriction was more prevalent in controls, suggesting that CR may be associated with melanoma status. The antitumor benefits of CR have been demonstrated in murine models for different tumor types, including breast, colon, liver, skin, as well as lung cancer [35]. In these models, CR has been shown to remodel the gut microbiota [36] and reduce levels of circulating growth factors and cytokines, vascular disturbances and inflammation levels. These changes may contribute to a reduction in tumor development and progression. Human studies have also found that CR lowers inflammatory and endocrine markers, with an inverse relationship observed between CR and breast cancer risk [37]. Additionally, CR has been linked to a decreased risk of non-melanoma skin cancer [38]. Given melanoma’s significant immunogenic component [39], dietary interventions that reduce systemic inflammation, modulate immune responses, and potentially affect tumor cell plasticity may in fact be biologically relevant.

In this study, control individuals, particularly men, reported skipping meals more frequently than melanoma patients. Interestingly, this pattern aligns with evidence suggesting that IF or fasting-mimicking diets may enhance immunotherapy efficacy. These effects have been linked to increased effector T-cell infiltration, suppression of regulatory T cells, and modulation of immune-related pathways. Such dietary interventions have also been shown to reduce myeloid-derived suppressor cell populations and promote a nutrient-poor tumor microenvironment that favors immune activation [40]. Nevertheless, in our cohort, these associations did not reach statistical significance, and only CR showed a significant relationship with melanoma risk. However, it is important to note that our dietary data reflects short-term intake patterns and may not capture sustained adherence to these dietary strategies. Longitudinal studies are needed to determine how sustained dietary behaviors and their biological effects relate to melanoma risk.

Regarding food restriction, we observed that limiting dairy products is associated with an increased risk of melanoma, consistent with previous research in other melanoma populations [14]. Several studies have described an antitumor mechanism similar to that of CR and IF mediated by probiotic agents, such as *Lactobacillus*, found in fermented dairy products [41]. It has been shown that an abundance of *Lactobacillus* in the intestinal microbiota not only protects against the appearance of toxicities associated with immunotherapy in melanoma [42] but also enhances its efficacy [43]. Furthermore, *Lactobacillus* has been found to mobilize host immune cells and promote immunoglobulin production, thereby stimulating the immune response against tumor proliferation [41]. The decrease in *Lactobacillus* in the intestinal microbiota due to dairy product restriction could explain the increased melanoma risk detected in our cohort.

Additionally, our data suggests that restriction of sugary products may also be associated with protection against melanoma, in line with previous evidence. Two case–control studies carried out in a Mediterranean population observed that the intake of products with a high glycemic load increased the risk of developing melanoma, although the exact pathophysiological mechanism remains unclear [32,44].

The World Health Organization (WHO) classifies red meat as “probable carcinogenic” and processed meat as “carcinogenic” [45] due to their association with an increased risk of various cancer types [46], mainly colorectal cancer [25,47]. In contrast, two previous studies showed an inverse relationship between the consumption of red meat and processed meat and the occurrence of melanoma, attributing this to the presence of potentially beneficial components in these foods [48]. In our cohort, we observed that a high intake of processed meat was associated with an increased risk of melanoma, but not with the consumption of red meat.

As to fish intake and melanoma risk, studies have been limited and yielded inconsistent results [12]. Research from the NIH-AARP Diet and Health Study identified a positive relationship between both total fish intake and specific types of fish consumption and melanoma risk [49]. However, other case–control studies have reported either a protective effect [13,33] or no significant association [50], underscoring the need for further research. Our results showed a tendency towards an association between white fish consumption and melanoma risk. One possible explanation may lie in the cooking methods commonly used by melanoma patients in our cohort, as they reported using unhealthy cooking practices more frequently than controls. Food prepared using unhealthy methods, such as frying, when subjected to high temperatures and/or large amounts of fat, accumulates genotoxic and carcinogenic components that damage DNA and contribute to cancer development [51]. Our findings are consistent with this effect in the case of the cooking of eggs, white fish and blue fish, which are consistent with studies on other cancer types [52]. However, the evidence regarding cooking methods and melanoma risk remains inconclusive. Some recent studies have found no carcinogenic risk associated with fish consumption regardless of cooking methods [53,54], while others have reported conflicting results. Li et al., for example, observed that non-fried fish intake was associated with increased melanoma risk, whereas fried fish intake showed an inverse relationship [49]. These discrepancies underscore the complexity of dietary exposures and the importance of considering both food type and preparation method in future research.

We did not observe statistically significant associations between other dietary components analyzed, including dietary fiber and fruit and vegetable intake. In our study, dietary intake was assessed only for the week preceding questionnaire administration, which may not reflect long-term consumption patterns necessary to elicit protective effects. This limitation could partly explain the discrepancy between our findings and those of prior studies. 

Finally, we also evaluated whether PA may influence the risk of developing melanoma, building on robust evidence linking PA to reduced incidence and improved outcomes in several cancers, including colorectal, endometrial, and breast cancers [55,56], and for its ability to mitigate treatment-related adverse effects such as fatigue, cardiovascular toxicity, and psychological distress [15,57]. The association between PA and melanoma risk has long been debated. Early studies reported a positive correlation, mainly attributed to increased UV exposure during outdoor exercise. However, recent research has challenged this view, suggesting that PA may have a protective role [15].

Our findings align with this evolving perspective, suggesting that PA may be associated with a lower melanoma risk, particularly in women. Interestingly, vigorous activity appears to confer protection regardless of gender, suggesting that exercise intensity may be a key factor. Both moderate and regular PA are known to modulate immune function and reduce inflammation [58], which may partly explain this association. A recent review on PA and its role in anticancer immunity supports this observation, suggesting that vigorous exercise may enhance anti-tumor immunity by increasing tumor-infiltrating natural killer (NK) cells, boosting its activity, and promoting lymphocyte proliferation across multiple cancer types [59,60]. Regular exercise can enhance NK cell cytotoxic activity against tumor cells and reshape the T-cell repertoire by potentially increasing the proportion of naive CD8+ T cells. In parallel, it can increase immune cell infiltrates in tumors, attenuate chronic systemic inflammation and increase gut microbiota diversity, which may further support antitumor immunity [60]. Preclinical models showed that PA slows tumor growth and remodels the tumor microenvironment by improving perfusion and oxygenation. These changes can normalize tumor vasculature, enhance intratumoral drug delivery, and facilitate the recruitment of effector immune cells, thereby potentially improving response to immunotherapy [17,57]. In addition, emerging evidence indicates that exercise-induced changes in the gut microbiota can further potentiate antitumor immunity [61], perhaps through microbial metabolites that enhance T-cell-mediated responses and improve the efficacy of immune checkpoint inhibitors in preclinical melanoma models. These biological mechanisms provide a plausible framework to interpret the inverse associations observed in our study. However, these findings should be interpreted with caution given that PA was assessed during the week following melanoma diagnosis, when treatment-related fatigue, mobility restrictions, or medical advice may have substantially reduced activity levels in patients independently of any true association with melanoma risk. Therefore, these results should be considered hypothesis-generating rather than conclusive evidence of a protective effect.

These findings may be partly explained by underlying biological mechanisms, including sex-related differences. The stronger protective effect observed in women may be partially explained by hormonal mechanisms. Sex-related differences in melanoma incidence and outcomes are well established and cannot be completely explained by difference in behavior. Therefore, sex hormones have been proposed to play a role in melanoma biology [62,63]. Estrogen has been implicated in melanoma development and progression through estrogen receptor signaling [62]. Mechanistically, it has been shown that estrogen can activate GPER-mediated pathways, leading to increased MITF activity, modulation of melanocytic differentiation programs, and enhanced immune recognition, while concomitantly suppressing oncogenic pathways such as c-Myc [62]. Beyond tumor-intrinsic effects, estrogen signaling may also reshape the tumor microenvironment by modulating immune responses and inflammatory pathways, further contributing to melanoma cell plasticity and disease progression [63]. Moreover, PA is known to reduce circulating levels of estrogens and growth factors, which could mitigate estrogen-driven tumorigenesis, particularly in premenopausal women, potentially contributing to the sex-specific differences observed in our cohort.

Emerging evidence also suggests that androgen receptor signaling pathways play a relevant role in the sex-differences observed in melanoma patients. Men, who generally show higher androgen receptor activity, often manifest more aggressive tumors, less favorable therapeutic responses, and poorer survival outcomes compared to women. In this vein, a recent *in vitro* study showed that sustained androgen receptor signaling may contribute to tumor aggressiveness by promoting its proliferation and invasiveness and its NK immune-escape [64]. Moreover, PA contributes to stress adaptation, and interacts with the tumor microenvironment, as well as promoting tumor plasticity [64,65,66]. These mechanisms may also be modulated by PA, which is known to influence systemic inflammation, hormone levels, and metabolic homeostasis, potentially interacting with sex-specific biological mechanisms, although these were not directly assessed in our analysis. Our data further suggests that the frequency of PA increases its beneficial effects, reinforcing the importance of sustained exercise. These observations underscore the importance of considering both biological sex and exercise patterns when evaluating lifestyle interventions for melanoma prevention and prognosis.

Taken together, lifestyle-related factors, such as intermittent fasting, nutrient composition and exercise, may influence melanoma development and progression by regulating metabolism, inflammation, antitumor immunity, and gut microbiota composition [40].

Importantly, gut microbiota, strongly shaped by dietary patterns, plays a modulatory role in regulating the immune system and metabolism. In recent years, growing evidence has linked gut microbiota composition to the development, prognosis, and treatment response to melanoma [67]. In particular, treatment response has frequently been associated with greater microbial diversity and enrichment of beneficial taxa such as *Akkermansia*, *Bifidobacterium*, *Ruminococcaceae*, and *Faecalibacterium* [40,68,69]. In this context, commensals produce microbial metabolites, particularly short-chain fatty acids, that mediate immunoregulatory functions by activating G protein-coupled receptors and inhibiting histone deacetylases, thereby promoting dendritic cell activation and enhancing the response of antitumor T cells. Beyond these systemic effects, tumor-resident microbial communities can also shape the local immune microenvironment by promoting cytotoxic T-cell infiltration and modulating cytokine signaling pathways that enhance antitumor immunity [68,70].

Notably, the relationship between gut microbiota and melanoma biology may also be shaped by biological sex. The gut microbiota is known to regulate circulating estrogen levels through the so-called “estrobolome”, a collection of bacterial genes encoding enzymes such as β-glucuronidases that deconjugate estrogens in the intestinal lumen, allowing their reabsorption into the bloodstream via enterohepatic circulation [71,72]. Disruptions in estrobolome function may therefore alter systemic estrogen availability and, consequently, estrogen-mediated effects on melanoma cell biology, immune modulation, and tumor microenvironment remodeling. A bidirectional relationship exists between sex hormones and gut microbiota composition where estrogens modulate microbial diversity, while the microbiota in turn regulates hormone bioavailability, creating sex-dependent crosstalk that may contribute to observed differences in melanoma risk and outcomes between men and women [72,73].

At the cellular level, these systemic and microenvironmental changes may converge and affect melanoma cell plasticity. This plasticity arises from dynamic metabolic, transcriptional, and epigenetic reprogramming that enables melanoma cells to adapt and survive under environmental stressors such as hypoxia, nutrient deprivation, and immune pressure [74,75]. For example, it has been shown that changes in the phenotype may be driven by changes in ROR1 and ROR2 expression levels involved in non-canonical Wnt5A pathway signaling. In hypoxic conditions, this induces a shift from ROR1-positive melanomas, which are highly proliferative and minimally invasive, toward a ROR2-positive phenotype, which is more invasive and less proliferative [76]. Beyond hypoxia-driven phenotypic switching, emerging evidence indicates that targeting calcium channels can further modulate melanoma cell stress responses. Specifically, pharmacological inhibition of calcium channels has been shown to increase intracellular reactive oxygen species levels in melanoma cells, which in turn upregulates the surface expression of NKG2D ligands of the ULBP family. Since ULBP proteins are recognized by NK cells and cytotoxic T lymphocytes, their increased membrane expression enhances immune recognition and susceptibility to immune-mediated killing, representing a potentially relevant interaction between metabolic stress, tumor plasticity, and antitumor immunity [77].

Ultimately, all of these processes converge on the tumor microenvironment and plasticity of melanoma cells, two key factors that determine tumor behavior, immune escape, and response to treatment.

### Limitations of the Study

This study has several limitations that should be considered when interpreting the results. First, the questionnaire was adapted from the PREDIMED study but was not formally validated, which may have introduced measurement errors. In addition, dietary intake and PA were assessed only during the week preceding questionnaire administration and relied on self-reported data, which may not accurately reflect long-term behavioral patterns and may be affected by recall bias, limiting the ability to extrapolate these findings to lifestyle habits. Second, the use of different recruitment strategies for melanoma patients and healthy individuals may have introduced selection bias and limited comparability between groups. Although an age and sex adjustment was performed, controls recruited through an online questionnaire may not be representative of the general population as education level, health awareness, or socioeconomic status were not systematically collected and their potential impact on our findings cannot be fully quantified. Differences in disease stage and treatment status at the time of questionnaire completion may have also influenced patients’ responses, particularly regarding PA, and may introduce reverse causation, as behaviors could have changed after diagnosis or treatment initiation. In addition, although different types of physical activity were collected, no information was available regarding whether these activities were performed indoors or outdoors, or on sun exposure or photoprotection habits. Therefore, the potential confounding effect of UV exposure on the observed associations could not be properly assessed. In addition, patient questionnaires were completed manually, whereas controls responded digitally with mandatory fields, resulting in a higher proportion of missing data among patients. Analyses were conducted using a complete-case approach, which may have reduced the effective sample size and introduced bias if data was not missing at random. Finally, no adjustment for potential confounders was performed due to the lack of systematically collected data on relevant variables, which may result in residual confounding.

## 5. Conclusions

Our findings suggest that dietary patterns and lifestyle factors may well be associated with melanoma risk. This study reinforces growing evidence that higher intakes of processed meats and sugary products are associated with a higher melanoma risk. Additionally, dairy product restriction was associated with increased melanoma risk, suggesting a potentially protective role for its consumption. These results also align with global nutritional recommendations and support lifestyle changes toward healthier cooking methods and regular PA, given their observed protective associations. Overall, although the results should be interpreted with caution, this study highlights potential relationships between diet, PA, and melanoma risk, supporting the need for further studies in larger cohorts. We can therefore conclude that combined dietary–lifestyle patterns may represent modifiable targets for melanoma prevention strategies.

## Figures and Tables

**Table 1:** Analysis of the demographic characteristics of the two cohorts.

Demographic Characteristics	Controls (n = 166)	Melanoma Patients (n = 130)	*p*-Value
Age at questionnaire completion ^1^ (median ± IQR)	56 ± 21	61 ± 23	0.047
Sex % (n)			0.558
Men	48.19 (80)	52.31 (68)	
Women	51.80 (86)	47.70 (62)	
BMI (kg/m^2^) (median ± IQR)	25.70 ± 6.21	26.17 ± 5.57	0.805
BMI categorized % (n)			0.648
Underweight	0.60 (1)	0.81 (1)	
Normal weight	42.17 (70)	38.21 (47)	
Overweight	33.73 (56)	40.65 (50)	
Obesity	23.49 (39)	20.33 (25)	
Missing values	0	7	

^1^ Age at questionnaire completion for all participants; in patients, the age at questionnaire completion also corresponds to age at melanoma diagnosis. BMI, body mass index.

**Table 2:** Evaluation of the impact of dietary habits and the intake of certain foods on the risk of developing melanoma.

**Meal or Certain Food Restrictions**					
**Type of Food**	**Controls** **(n = 166)** **% (n)**	**Melanoma Patients** **(n = 130)** **% (n)**	**OR (CI 95%)**	***p*** **-Value**	**Adjusted *p*-Value (FDR)**
Omission of any meal	24.7 (41)	15.6 (20)	0.57 (0.31–1.02)	0.057	0.091
Men	31.3 (25)	14.9 (10)	0.39 (0.17–0.88)	**0.021**	0.075
Women	18.6 (16)	16.4 (10)	0.86 (0.36–2.05)	0.729	0.729
Missing values	0	2	*-*	*-*	*-*
All food	51.2 (85)	29.9 (38)	0.41 (0.25–0.66)	**<0.001**	**<0.001**
Men	43.8 (35)	26.2 (17)	0.46 (0.22–0.92)	**0.028**	0.075
Women	58.1 (50)	33.9 (21)	0.37 (0.19–0.73)	**0.004**	**0.016**
Missing values	0	3	*-*	*-*	*-*
Dairy Products	8.4 (14)	19.7 (25)	2.66 (1.32–5.36)	**0.005**	**0.013**
Men	8.8 (7)	16.9 (11)	2.12 (0.77–5.84)	0.138	0.221
Women	8.1 (7)	22.6 (14)	3.29 (1.24–8.73)	**0.013**	**0.035**
Missing values	0	3	*-*	*-*	*-*
Meat and Fish	12 (20)	15.7 (20)	1.36 (0.70–2.66)	0.361	0.361
Men	8.8 (7)	13.8 (9)	1.68 (0.59–4.78)	0.330	0.440
Women	15.1 (13)	17.7 (11)	1.21 (0.5–2.92)	0.670	0.729
Missing values	0	3	*-*	*-*	*-*
Sugary Products	33.1 (55)	5.5 (7)	0.12 (0.05–0.27)	**<0.001**	**<0.001**
Men	31.3 (25)	6.2 (4)	0.14 (0.05–0.44)	**<0.001**	**<0.001**
Women	34.9 (30)	4.8 (3)	0.1 (0.03–0.33)	**<0.001**	**<0.001**
Missing values	0	3	*-*	*-*	*-*
Cereals	3 (5)	5.5 (7)	1.88 (0.58–6.06)	0.285	0.326
Men	5 (4)	3.1 (2)	0.6 (0.11–3.4)	0.560	0.643
Women	1.2 (1)	8.1 (5)	7.46 (0.85–65.5)	**0.036**	0.072
Missing values	0	3	*-*	*-*	*-*
Processed Products	10.8 (18)	6.3 (8)	0.55 (0.23–1.32)	0.175	0.233
Men	5 (4)	6.2 (4)	1.25 (0.3–5.18)	0.762	0.762
Women	16.3 (14)	6.5 (4)	0.36 (0.11–1.14)	0.071	0.114
Missing values	0	3	-	-	-
**Consumption of Different Foods and Micronutrients More Than Three Days a Week**					
**Type of Food**	**Controls** **(n = 166)** **% (n)**	**Melanoma Patients** **(n = 130)** **% (n)**	**OR (CI 95%)**	***p*** **-Value**	**Adjusted *p*-Value (FDR)**
Red Meat	45.2 (75)	48.5 (63)	1.14 (0.72–1.81)	0.574	0.835
Men	53.8 (43)	54.4 (37)	1.03 (0.54–1.97)	0.940	0.951
Women	37.2 (32)	41.9 (26)	1.22 (0.63–2.38)	0.560	0.733
Processed meats	33.7 (56)	56.2 (73)	2.52 (1.57–4.04)	**<0.001**	**<0.001**
Men	40 (32)	57.4 (39)	2.02 (1.05–3.89)	**0.035**	0.358
Women	27.9 (24)	54.8 (34)	3.14 (1.58–6.24)	**0.001**	**0.021**
White Fish	33.7 (56)	46.9 (61)	1.74 (1.08–2.78)	**0.021**	0.133
Men	27.5 (80)	41.2 (28)	1.85 (0.93–3.67)	0.080	0.394
Women	39.5 (34)	53.2 (33)	1.74 (0.9–3.37)	0.099	0.448
Blue Fish	40.4 (67)	49.2 (64)	1.43 (0.90–2.28)	0.127	0.365
Men	38.8 (31)	51.5 (35)	1.68 (0.87–3.23)	0.121	0.417
Women	41.9 (36)	46.8 (29)	1.22 (0.63–2.36)	0.550	0.733
Fiber-poor Cereals	82.5 (137)	81.5 (106)	0.94 (0.51–1.7)	0.825	0.960
Men	88.8 (80)	88.2 (60)	0.95 (0.35–2.62)	0.922	0.951
Women	76.7 (66)	74.2 (46)	0.87 (0.41–1.86)	0.721	0.883
Fiber-rich Cereals	56 (93)	43.1 (56)	0.59 (0.37–0.94)	**0.027**	0.133
Men	50 (40)	36.8 (25)	0.58 (0.30–1.12)	0.106	0.417
Women	61.6 (53)	50 (31)	0.62 (0.32–1.21)	0.159	0.509
F&V rich in Phenols and Antioxidants	92.8 (154)	93.1 (121)	1.05 (0.43–2.57)	0.919	0.990
Men	88.8 (71)	92.6 (63)	1.6 (0.51–5.02)	0.419	0.729
Women	96.5 (83)	93.5 (58)	0.52 (0.11–2.43)	0.402	0.653
F&V rich in Carotenoids	81.9 (136)	91.5 (119)	2.39 (1.15–4.97)	**0.018**	0.133
Men	75 (60)	86.8 (59)	2.19 (0.92–5.19)	0.072	0.394
Women	88.4 (76)	96.8 (60)	3.95 (0.83–18.7)	0.065	0.448
F&V rich in Potassium	91.6 (152)	91.5 (119)	0.99 (0.44–2.27)	0.990	0.990
Men	91.3 (73)	92.6 (63)	1.12 (0.36–3.4))	0.756	0.853
Women	91.9 (79)	90.3 (56)	0.83 (0.26–2.59)	0.744	0.883
F&V rich in Vitamin E	96.4 (160)	90 (117)	0.34 (0.13–0.91)	**0.026**	0.133
Men	95 (76)	88.2 (60)	0.39 (0.11–1.37)	0.133	0.434
Women	97.7 (84)	91.9 (57)	0.27 (0.05–1.45)	0.105	0.448
F&V rich in Vitamin C	88 (146)	85.4 (111)	0.8 (0.41–1.57)	0.517	0.769
Men	83.8 (67)	86.8 (59)	1.27 (0.51–3.19)	0.607	0.814
Women	91.9 (79)	83.9 (52)	0.46 (0.17–1.29)	0.133	0.451

Bold values indicate statistically significant results. F&V, fruits and vegetables; OR, odds ratio; CI 95%, 95% confidence interval; FDR, false discovery rate.

**Table 3:** Evaluation of the impact of unhealthy cooking methods on the risk of melanoma. ‘Unhealthy cooking’ is understood as cooking primarily through frying and breading.

Type of Food	Controls (n = 166) % (n)	Melanoma Patients (n = 130) % (n)	OR (CI 95%)	*p*-Value	Adjusted *p*-Value (FDR)
Egg	30.1 (49)	49.1 (57)	2.25 (1.37–3.69)	**0.001**	**0.006**
Men	32.5 (26)	60 (39)	3.12 (1.58–6.16)	**0.001**	**0.006**
Women	27.7 (23)	35.3 (18)	1.42 (0.67–3.01)	0.355	0.840
Missing values	3	14	*-*	*-*	*-*
White Meat	3.7 (6)	8.3 (10)	2.35 (0.83–6.65)	0.099	0.119
Men	5.1 (4)	12.3 (8)	2.6 (0.75–9.05)	0.123	0.148
Women	2.4 (2)	3.6 (2)	1.53 (0.21–11.18)	0.684	0.957
Missing values	5	10	-	-	*-*
Red Meat	2 (3)	6 (6)	3.19 (0.78–13.07)	0.090	0.119
Men	1.3 (1)	8.9 (5)	7.26 (0.82–63.95)	**0.040**	0.080
Women	2.6 (2)	2.3 (1)	0.88 (0.08–10.03)	0.921	0.957
Missing values	13	30	*-*	*-*	*-*
White Fish	7.5 (12)	17.1 (20)	2.54 (1.19–5.44)	**0.014**	**0.028**
Men	6.3 (5)	20.6 (13)	3.85 (1.29–11.47)	**0.011**	**0.033**
Women	8.6 (7)	13 (7)	1.57 (0.52–4.78)	0.420	0.840
Missing values	6	13	*-*	*-*	*-*
Blue Fish	9.6 (15)	23.9 (26)	2.95 (1.47–5.88)	**0.002**	**0.006**
Men	15.6 (12)	27.4 (17)	2.05 (0.89–4.7)	0.088	0.132
Women	3.8 (3)	19.1 (9)	6 (1.54–23.46)	**0.005**	**0.030**
Missing values	10	21	*-*	*-*	*-*
Seafood	3.4 (5)	3.9 (3)	1.17 (0.27–5.02)	0.835	0.835
Men	4.3 (3)	5 (2)	1.18 (0.19–7.35)	0.863	0.863
Women	2.5 (2)	2.7 (1)	1.07 (0.94–12.18)	0.957	0.957
Missing values	17	53	*-*	*-*	*-*

Bold values indicate statistically significant results. OR, odds ratio; CI 95%, 95% confidence interval; FDR, false discovery rate.

**Table 4:** Evaluation of the impact of physical activity intensity and frequency on the risk of developing melanoma. “General Physical Activity” is defined as engaging in any intensity of exercise including moderate and/or vigorous activity.

**General Physical Activity Practice and Its Intensity in the Last Seven Days**					
**Intensity of Physical Activity**	**Controls** **(n = 166)** **% (n)**	**Melanoma Patients** **(n = 130)** **% (n)**	**OR (CI 95%)**	***p*** **-Value**	**Adjusted *p*-Value (FDR)**
General Physical Activity	70.5 (117)	60.2 (71)	0.63 (0.39–1.04)	0.07	0.082
Men	73.8 (59)	72.6 (45)	0.94 (0.45–1.99)	0.876	0.947
Women	67.4 (58)	46.4 (26)	0.42 (0.21–0.84)	**0.013**	**0.029**
Missing values	(0)	(12)	*-*	*-*	*-*
Moderate Intensity	68.1 (113)	57.5 (69)	0.64 (0.39–1.03)	0.067	0.082
Men	71.3 (57)	68.3 (43)	0.87 (0.42–1.78)	0.698	0.947
Women	65.1 (56)	45.6 (26)	0.45 (0.23–0.891)	**0.021**	**0.029**
Missing values	(0)	(10)	-	-	*-*
Vigorous Intensity	38.6 (64)	15.5 (17)	0.29 (0.16–0.53)	**<0.001**	**<0.001**
Men	45 (36)	23.2 (13)	0.37 (0.17–0.79)	**0.009**	**0.032**
Women	32.6 (28)	7.4 (4)	0.17 (0.05–0.51)	**0.001**	**0.004**
Missing values	(0)	(20)	*-*	*-*	*-*
**Frequency of General Physical Activity Practice in the Last Seven Days**					
**Frequency of Physical Activity**	**Controls** **(n = 166)** **% (n)**	**Melanoma Patients** **(n = 130)** **% (n)**		***p*** **-Value**	**Adjusted *p*-Value (FDR)**
All				0.142	0.232
0–1 days	29.5 (49)	40.9 (47)			
2–3 days	19.3 (32)	15.7 (18)			
>3 days	51.2 (85)	43.5 (50)			
Missing values	0	15			
Men				0.713	0.909
0–1 days	26.3 (21)	28.8 (17)			
2–3 days	18.8 (15)	13.6 (8)			
>3 days	55 (44)	57.6 (34)			
Women				**0.033**	**0.045**
0–1 days	32.6 (28)	53.6 (30)			
2–3 days	19.8 (17)	17.9 (10)			
>3 days	47.7 (41)	28.6 (16)			

Bold values indicate statistically significant results. OR, odds ratio; CI 95%, 95% confidence interval; FDR, false discovery rate.

## Data Availability

The data presented in this study are available on request from the corresponding author due to the inclusion of sensitive participant-reported information that could compromise individual privacy.

## Supplementary Materials

The following supporting information can be downloaded at https://www.mdpi.com/article/10.3390/nu18121919/s1. Material S1: Questionnaire on dietary habits and physical activity.

## Abbreviations

The following abbreviations are used in this manuscript:

AICR	American Institute for Cancer Research
BMI	Body mass index
CR	Calorie restriction
IF	Intermittent fasting
MD	Mediterranean diet
NK	Natural killer
OR	Odds ratio
PA	Physical activity
UVR	Ultraviolet radiation
WCRF	World Cancer Research Fund
WHO	World Health Organization
