# Supplementary material for: The Influence of Dietary and Physical Exercise Habits on Melanoma Risk: A Case–Control Study
Source: Nutrients. 2026 Jun 12;18(12):1919. doi: 10.3390/nu18121919 (PMC13306214; doi:10.3390/nu18121919)
Supplement: Supplementary file 1 [file nutrients-18-01919-s001.zip › nutrients-4347616-Material S1.pdf]

Annex 1. Questionnaire on dietary habits and physical activity.

Name and Surname: ____

Date: _________________

Age: _______years **Sex:** MALE FEMALE

Weight: _______Kg **Height:** _______cm

DIET

1. How many **days per week** do you skip breakfast, lunch, or dinner?
   a. None
   b. 1–2 days
   c. 3–5 days
   d. 6–7 days

2. Do you restrict or avoid the intake of any type of food or food groups (for example, meat or dairy products)?
   a. Yes
   b. No

   If you answered YES, please explain which foods or types of foods you avoid:

____

____

____

3. Do you follow a **vegetarian** diet?
   a. Yes
   b. No

4. Please mark with an X the foods from each group that you consume **at least 3 times per week.**

Meat and Fish		Fruits		Vegetables		Grains		Dairy and Fats	
Eggs		Apples		Potato		Rice		Cow's milk	
Beef		Oranges		Tomato		Brown rice		Soy milk	
Lamb		Pineapple		Carrot		Pasta		Yogurt	
Pork		Grapes		Onion		Whole-wheat pasta		Aged cheese	
Rabbit		Kiwi		Garlic		White bread		Fresh cheese	
Chicken		Strawberries		Broccoli		Whole-grain bread		Olive oil	
Turkey		Peach		Cauliflower		Multigrain bread		Extra virgin olive oil	
White meat (all)		Plantains		Pumpkin		Couscous		Sunflower oil	
Red meat (all)		Melon		Zucchini		Semolina		Butter	
Frankfurters and sausages		Watermelon		Cucumber		Wheat flour		Margarine	
Processed meats		Bananas		Lettuce		Oat flour		Other dairy or fats (specify below)	
Hake		Plums		Green beans		Cereals			
Sole		Pears		Swiss chard		Other grains (specify below)			
Monkfish		Coconut		Spinach					
Sardines		Cherries		Lentils					
Salmon		Almonds		Beans					
Tuna		Walnuts		Chickpeas					
White fish (all)		Hazelnuts		Mushrooms					
Blue fish (all)		Other fruits (specify below)		Other vegetables (specify below)					

Seafood									
Other meats and fish (specify below)									

Write down any other foods you consume frequently that are not included in the list above:

____

____

____

5. Mark with an X your **weekly** consumption of the following foods

How many times per week do you consume the following foods:	Never	Monthly	1-2	3-4	5-7	8-10	11 +
Mineral water							
Coffee or tea							
Sugary drinks (e.g., Coca-Cola)							
Energy drinks (e.g., Red Bull)							
Packaged juice							
Isotonic drinks							
Fast food							
Sausages, frankfurers, bacon							
Processed meats / cold cuts							
French fries or similar							
Sweet cereals, cereal bars							
White bread / corn bread							

Snacks							
Cakes, muffins, donuts, etc.							
Candies and sweets							
Creamy cheeses							
Creamy sauces (e.g., mayonnaise)							
Canned fruits and vegetables							
Fresh fruits and vegetables							
Pasta, rice							
White meat (e.g., chicken, turkey, rabbit, pork)							
Red meat (e.g., beef, lamb)							
White fish (e.g., hake, monkfish, sole)							
Blue fish (p.e. sardines, tuna, salmon)							
Nuts							
Olive oil							
Yogurt							

6. How often do you take multivitamin supplements or folic acid **per week**?
    a. Never
    b. 1–2 days
    c. 3–5 days
    d. 6–7 days

7. Do you take any other vitamin or mineral supplements (iron, calcium, etc.)?
    a. Yes
    b. No

    If you answered YES, please specify which ones:

____

____

____

____

8. Mark with an X the cooking method you use **most frequently** for the following food groups:

	Raw	Boiled	Grilled / Pan-seared	Baked / Oven-cooked	Stewed	Fried	Battered / Breaded
Vegetables							
Egg							
White meat							
Red meat							
White fish							
Blue fish							
Seafood							

9. Mark with an X **all** the cooking method you use for the following food groups:

	Raw	Boiled	Grilled / Pan-seared	Baked / Oven-cooked	Stewed	Fried	Battered / Breaded
Vegetables							
Egg							
White meat							
Red meat							
White fish							
Blue fish							
Seafood							

PHISICAL ACTIVITY

1. **In the last 7 days,** on how many days did you perform moderate physical activity (e.g., walking, dancing, cycling…) **for at least 60 minutes?**
   a. 0-1 days
   b. 2-3 days
   c. 4-5 days
   d. 6-7 days

2. **In the last 7 days,** on how many days did you perform vigorous physical activity (e.g., running, spinning, swimming…) **for at least 30 minutes?**
   a. 0-1 days

b. 2-3 days
c. 4-5 days
d. 6-7 days

3. Outside of school or work, how many **hours per day** do you spend watching television, browsing the Internet, or playing video games?
   a. 0 -1 hours
   b. 2-3 hours
   c. 4-5 hours
   d. 6+ hours

4. In the table below, mark with an X the number of **weekly hours** you spend performing any of the following activities (during both summer and winter months).

**SUMMER**	0-1	2-3	4-5	6-7	8+
Walking					
Cycling					
Running					
Climbing stairs					
Aerobics, dancing					
Gym / fitness training					
Vigorous activity (sweating, increased heart rate)					
House cleaning					
Gardening					
OTHER					

If you selected OTHER, please specify:

____

____

____

**WINTER**	0-1	2-3	4-5	6-7	8+
Walking					

Cycling					
Running					
Climbing stairs					
Aerobics, dancing					
Gym / fitness training					
Vigorous activity (sweating, increased heart rate)					
House cleaning					
Gardening					
OTHER					

If you selected OTHER, please specify:

____

____

____

THANK YOU FOR YOUR COLABORATION!
